# P-298. Treatment of Carbapenem-Resistant Gram Negative Bacteremia based on Polymixin MIC

**DOI:** 10.1093/ofid/ofae631.501

**Published:** 2025-01-29

**Authors:** Sruthi Menon, S Lavanya, Ajay Anthur Nair, Thomas Babu, A Murali, Krishna S Nair

**Affiliations:** PSG IMSR and Hospitals, Coimbatore, Tamil Nadu, India; PSG IMSR and Hospitals, Coimbatore, Tamil Nadu, India; PSG IMSR and Hospitals, Coimbatore, Tamil Nadu, India; PSG IMSR and Hospitals, Coimbatore, Tamil Nadu, India; PSG IMSR and Hospitals, Coimbatore, Tamil Nadu, India; PSG IMSR and Hospitals, Coimbatore, Tamil Nadu, India

## Abstract

**Background:**

Recent studies from India have noted increasing resistance against ceftazidime- avibactam in enterobacteriacea and non-fermenters and hence, polymixins are mainstay in the treatment of carbapenem-resistant gram negative (CRGN) infections. There is no susceptible breakpoint for polymixins in view of concerns with clinical outcome and therefore, guidelines recommend combination therapy. However, studies have failed to show the superiority of polymixin combination therapy over monotherapy. Invitro data shows ≥ 90% PK-PD target attainment for isolates with colistin MIC ≤ 0.5 mg/L, but lower success rates in isolates with higher MICs. Aim of the study is to evaluate if the choice of treatment of CRGN infections can be guided by polymixin MIC.

**Figure:**
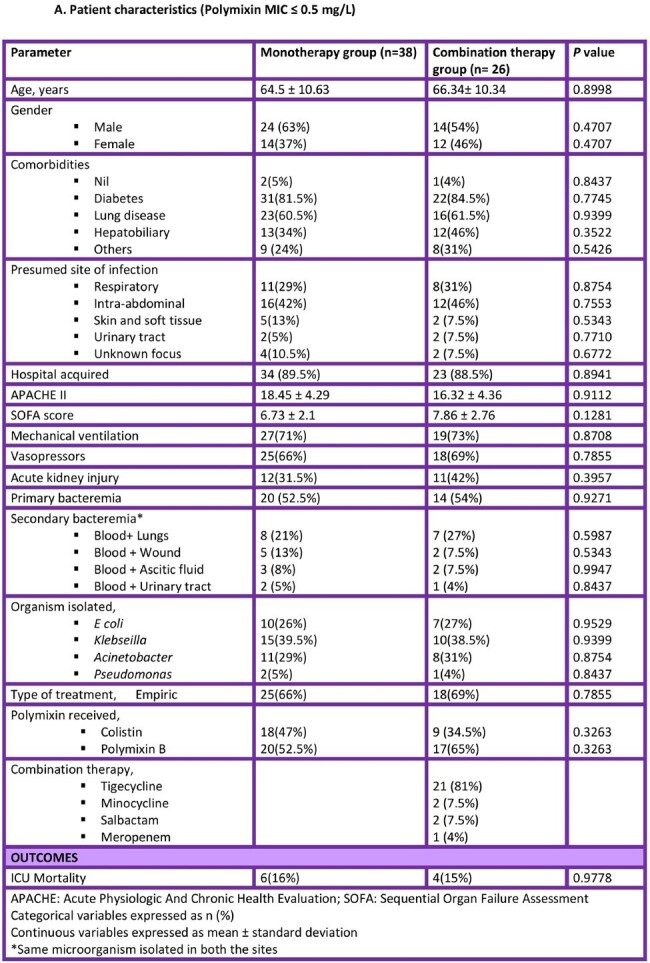

**Methods:**

We retrospectively evaluated critically-ill patient with CRGN bacteremia with intermediate in vitro susceptibility to colistin (MIC ≤ 2 mg/L; as per CLSI) at a tertiary care hospital in India from January 2022- June 2023. Patients who received treatment for the CRGN bacteremia for <48 h were excluded. Based on polymixin MIC, patients were grouped into two study groups: those that received polymixin monotherapy and those that received polymixin in combination with another agent susceptible in vitro. Chi-square test was used for categorical data and student’s t-test for continuous data. Analysis was performed using SPSS v28 and statistical significance was defined as p < 0.05.

**Figure:**
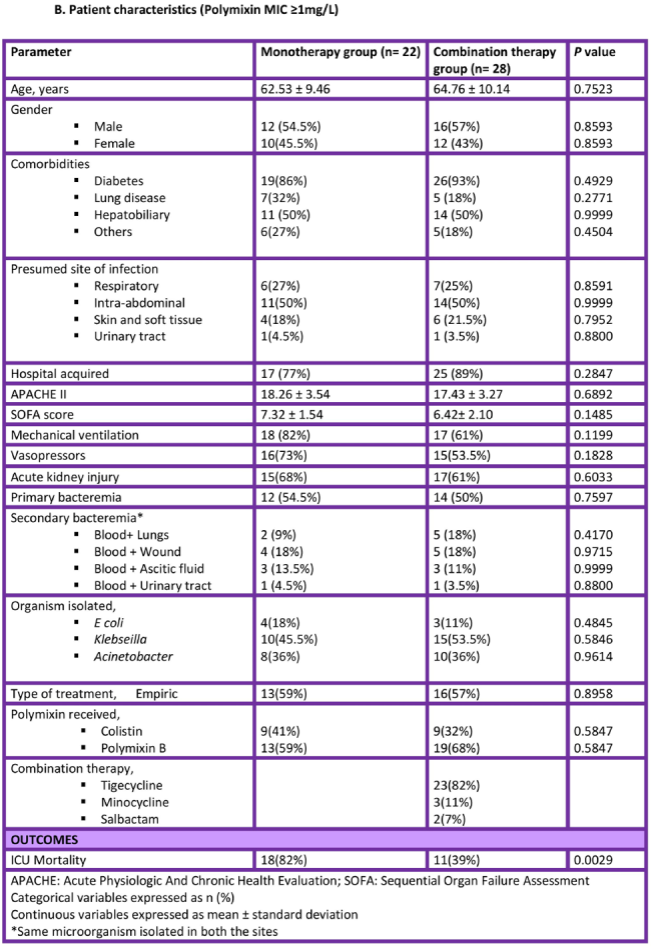

**Results:**

Of the 114 patients with CRGN bacteremia, 64 (56%) had an MIC of ≤ 0.5 and 50 (44%) had MIC ≥ 1. There was no statistically significant difference in the ICU mortality between monotherapy and combination therapy arms (16% vs 15%; *P* = 0.9778) for polymixin MIC ≤ 0.5. However, when the MIC was ≥ 1, a higher mortality was observed in the polymixin monotherapy arm (82% vs 39%; *P* = 0.0029).

**Conclusion:**

Isolates with MIC ≤ 0.5 can be treated with polymixin monotherapy alone while isolates with MIC ≥ 1 require a combination therapy of polymixin with another antibiotic suseptible invitro. However, this is a single centre retrospective study and further clinical trials are required to ascertain the feasibility of polymixin MIC guided treatment of CRGN infections.

**Disclosures:**

**All Authors**: No reported disclosures

